# Ultra-Fast-Track Extubation Within Four Hours Versus Conventional Ventilation After Robotic Coronary Artery Bypass Grafting: A Systematic Review of Intensive Care Unit Stay and Postoperative Atrial Fibrillation Outcomes

**DOI:** 10.7759/cureus.101621

**Published:** 2026-01-15

**Authors:** Muhammad Farhan, Tirath Patel, Dania Almarouj, Vindhesh Dixit, Mohammed Abdullah, Lamia Bnaian, Reem Imadeldin Ahmed Hamad, Elwy Josey, Aiswariya Anna Alexander, Ashfaq Ahmad, Adekunle E Omole

**Affiliations:** 1 Department of Medicine, Ajman University, Ajman, ARE; 2 Department of Neurological Surgery, California Institute of Behavioural Neurosciences and Psychology, Fairfield, USA; 3 College of Medicine, Al-Farabi Kazakh National University, Almaty, KAZ; 4 College of Medicine, Tbilisi State Medical University, Tbilisi, GEO; 5 College of Medicine, Al Qassimi Hospital, Sharjah, ARE; 6 College of Medicine, Gomal Medical College, Dera Ismail Khan, PAK; 7 Department of Anatomical Sciences, School of Engineering Medicine, Texas A&amp;M University School of Engineering Medicine (EnMed), Houston, USA

**Keywords:** early extubation protocols, enhanced recovery after surgery, icu length of stay, minimally invasive cardiac surgery, postoperative atrial fibrillation, robotic coronary artery bypass, thoracic epidural analgesia, ultra-fast-track extubation

## Abstract

Robotic-assisted coronary artery bypass grafting (RA-CABG) is a minimally invasive procedure that can lead to quicker recovery. Ultra-fast-track extubation (UFTE) within four hours post-surgery is a key element of enhanced recovery. This review compared UFTE versus conventional ventilation (>4 hours) on intensive care unit (ICU) stay and postoperative atrial fibrillation (POAF) in adults undergoing RA-CABG.

Following the Preferred Reporting Items for Systematic Reviews and Meta-Analyses (PRISMA) guidelines, databases (MEDLINE, Embase, Cochrane Central, and others) were searched until July 2025. Inclusion criteria targeted adults undergoing RA-CABG, with outcomes assessed by ICU length of stay (LOS) and POAF incidence. The risk of bias was assessed using the Cochrane Risk of Bias 2 (ROB 2) tool for randomized controlled trials (RCTs) and the Risk Of Bias In Non-randomized Studies of Interventions (ROBINS-I) tool for observational studies.

Nine studies (one RCT and eight retrospective cohorts; total n=3,223) were included. UFTE reduced ICU LOS (e.g., median 21 vs. 45 hours; p=0.001) and hospital LOS (1-4 days shorter). POAF rates were similar or lower with UFTE (e.g., 3.2% vs. 14%; p=0.004). No increases in mortality, re-intubation, or other complications occurred.

UFTE after RA-CABG safely shortened ICU and hospital stays without elevating POAF or adverse outcomes. These findings support the use of UFTE in enhanced recovery protocols for carefully selected patients. However, the observational nature of most evidence highlights the need for more high-quality RCTs to confirm benefits.

## Introduction and background

Coronary artery bypass grafting (CABG) is one of the cornerstones used in the treatment of multivessel coronary artery disease, with survival and symptomatic benefits over medical therapy and percutaneous interventions in specific populations [1,2]. Although surgical interventions have improved, conventional CABG through median sternotomy is characterized by a high morbidity, prolonged mechanical ventilation, and prolonged duration of intensive care unit (ICU) and hospital stay, all of which lead to increased healthcare expenses and prolonged functional recovery [3].

Minimally invasive cardiac surgery (MICS), as well as robotic-assisted CABG (RA-CABG), has been established to reduce the postoperative trauma with reduced surgical incisions, tissue trauma, and to facilitate a quicker recovery [4]. Robotics technology enables entirely endoscopic grafting (primarily to the left anterior descending artery, or LAD) without a sternotomy, which may be associated with reduced pain, decreased blood loss, and fewer infections [5]. Nevertheless, RA-CABG surgery is characterized by longer operative duration and complicated intraoperative practices, including one-lung ventilation, which leads to unique anesthetic challenges [6].

It is stated that early extubation and fast-tracking anesthesia interventions have a positive impact on cardiac surgery, resulting in a shorter duration of mechanical ventilation, a shorter stay in the ICU, and more efficient resource allocation without jeopardizing patient safety [7]. Fast-track elements specific to disease considerations include management of analgesia, implementation of goal-directed hemodynamics, early mobilization principles, as well as those focused on enhanced recovery after surgery (ERAS) programs [8,9]. Recent studies have found that fast-track extubation, commonly defined as occurring within six hours postoperatively, reduces postoperative complications and the length of stay (LOS) in conventional cardiac surgery [10,11].

Another optimization method aimed at achieving the end-of-the-day objective of faster recovery is ultra-fast-track extubation (UFTE), which involves early extubation in the operating room (OR) or within four hours post-surgery, with the intention of preventing further pulmonary complications and improving patient satisfaction [12]. While these protocols have demonstrated safety and efficacy in patients undergoing conventional cardiac surgery, their role in those receiving RA-CABG is less clear. The unique physiological challenges of the robotic procedure, including single-lung ventilation, prolonged anesthesia time, and a constrained surgical field, may influence both the feasibility and outcomes of UFTE in this population [13].

Postoperative atrial fibrillation (POAF) has been one of the most significant complications and frequent problems of cardiac surgery [14]. It is linked to a longer ICU and hospital stay, a higher risk of stroke, and high mortality [15]. It has been believed that ventilatory strategy, anesthetic depth, opioid use, and systemic inflammation are factors that cause POAF [16]. Earlier extubation and decreased sedation can potentially mitigate sympathetic activation and inflammation, thereby reducing the probability of arrhythmia [17].

Although UFTE has theoretical benefits after RA-CABG, airway safety, respiratory complications, and hemodynamic stability remain concerns [18]. The choices of the best patient, the anesthetic combinations, and the surgery bundles remain a matter of research. Currently, the literature on UFTE following RA-CABG is heterogeneous and scarce, which does not allow for making conclusive recommendations.

Considering the growing popularity of robotic-assisted cardiac surgery worldwide, the effectiveness and safety of UFTE should be thoroughly examined. This systematic review tested the hypothesis that UFTE (0-4 hours postoperative) compared to standard extubation (>4 hours) among adults undergoing RA-CABG can affect ICU LOS, the incidence of POAF, and other clinically relevant outcomes.

## Review

Methodology

This systematic review was conducted to determine the effects of UFTE versus traditional ventilation in patients undergoing RA-CABG. To maintain complete transparency, we adhered to a prospectively registered protocol in the PROSPERO (CRD420251169875) global database, specifying methodological pre-specifications, inclusion criteria, and analytic procedures. During the review process, we adhered to the guidelines of the Preferred Reporting Items for Systematic Reviews and Meta-Analyses (PRISMA) [19], thereby enhancing the clarity, reliability, and discernibility of our results.

Eligibility Criteria and Study Design

The studies met the inclusion criteria in that they involved the enrolment of adult patients (at least 18 years old) who underwent RA-CABG, including totally endoscopic coronary artery bypass (TECAB), minimally invasive direct coronary artery bypass (MIDCAB), or hybrid operations. The cases of MICS that utilized robotic assistance and reported information on robotic CABG patients were also considered. The intervention of interest was UFTE, which is the extubation conducted in the OR within the immediate period after the procedure or within four hours of admission. Some upgraded protocols adopted were multimodal analgesia, opioid sparing strategies, and regional anesthesia protocols like thoracic epidural or fascia plane blocks. The control groups included patients who were handled according to traditional extubation guidelines, in which they were extubated after more than four hours had elapsed, mostly in the ICU during regular postoperative care.

The primary outcome was ICU LOS, either in hours or days. Secondary outcomes included incidence of atrial fibrillation postoperatively (POAF) in the five days after operative procedures, LOS in the hospital, re-intubation rates, perioperative complications (e.g., stroke, pneumonia), blood transfusion requests, and mortality rate within 30 days. To enable the comparison, at least ICU LOS or POAF statistics had to be reported to qualify. Randomized controlled trials (RCTs), prospective and retrospective comparative cohort studies, as well as propensity-matched studies, were included where there were at least ten patients per arm of the study, as these methods are suitable for providing strong and substantive comparisons. To avoid disruptive data, RA-CABG data, non-comparative case series, abstracts, and studies without disaggregated RA-CABG data were excluded.

Sources of Information and Search Strategy

To locate the studies of interest, we conducted extensive searches of various databases, including MEDLINE, Embase, Cochrane Central Register of Controlled Trials (CENTRAL), CINAHL, Web of Science, Scopus, and ClinicalTrials.gov. We further searched in the International Clinical Trials Registry Platform (ICTRP) site of the World Health Organization and undertook a review of relevant conference proceedings. We searched all available records up to July 31, 2025, without any language limitations that would have narrowed our search and reduced the likelihood of retrieving all eligible literature. We have employed a search strategy designed in collaboration with a medical information specialist, utilizing controlled vocabulary terms (Medical Subject Headings (MeSH)) and topical text words to capture the ideas and concepts related to RA-CABG and fast-track/UFTE. In cases where the MEDLINE search strategy was used, controlled vocabulary and keywords were applied in conjunction with robotic-assisted CABG and fast-track extubation, which included the terms robotic coronary bypass, MICS, fast-track, ultra-fast-track, early extubation, and rapid weaning (see Appendices). All the identified citations were processed using EndNote software (Clarivate, London, UK) to perform de-duplication and then uploaded to the Rayyan platform (Rayyan Systems Inc., Cambridge, MA, US) for systematic screening.

Study Selection Process and Data Extraction

The screening process was rigorous for reviewers and commenced with titles and abstracts to select potentially relevant studies. The individuals deemed qualified were then subjected to a full-text review, which was conducted independently and in duplicate. We addressed any issues of disagreement regarding the inclusion of a study through a consensus discussion or adjudication by senior reviewers. We carefully documented the reasons behind our exclusion at each step during this process to maintain transparency and provide accurate reporting in the PRISMA flow diagram. Reviewers independently and in duplicate extracted the data using a standardized, pilot-tested spreadsheet. We gathered all the information such as the characteristics of study, characteristics of participants (demographic data on particular population, demographic data on healthy participants, demographic data on terminally ill participants), characteristics of surgery, characteristics of intervention, characteristics of comparator, and characteristics of clinical outcomes (ICU LOS, POAF, hospital LOS, re-hospitalization, 30-day mortality, adverse event). In instances where the results were reported as medians and ranges or interquartile ranges, we recoded these results to approximate means and standard deviations using validated statistical methods to enable homogeneous synthesis. Whenever discrepancies were found in the extracted data, we would reach an agreement or consult senior reviewers to ensure accuracy.

Risk of Bias Assessment

We used the necessary tools to independently determine the quality of the methodology and risk of bias in each of the included studies. In the case of RCTs, we used the Cochrane Risk of Bias 2 (RoB 2) [20] tool to assess the areas of randomization procedure, allocation concealment, blinding, outcome measurement, and reporting. In observational cohort studies, we have adopted the Risk Of Bias In Non-randomized Studies of Interventions (ROBINS-I) [21] tool to evaluate bias in the areas of confounding, sampling, categorization of interventions, non-adherence to planned interventions, data loss, outcome measurement, and selective reporting. Any disputes that were not resolved were thoroughly discussed by all reviewers, with a specific emphasis on areas that may have influenced the decision regarding bias.

Data Synthesis and Analysis

We determined that a quantitative meta-analysis was not appropriate due to the clinical and methodological heterogeneity of the included studies, which varied in research design, subjects, surgical methods, intervention procedures, and outcome measures across the reports. Instead, we conducted a more comprehensive qualitative synthesis, which narratively described our results and placed them in context. For continuous outcomes where continuous measures are defined, such as ICU and hospital LOS, we summarized reporting using descriptive methods with a focus on the direction and magnitude of effects between the intervention and comparator groups. Dichotomous results, such as POAF incidence and POAF mortality, were reported in counts and percentages, and commentary on statistical significance was provided where investigators reported such data. We prepared subgroup analyses to investigate heterogeneity related to the study design (RCTs vs. observational studies), extubation timing and setting (OR vs. ICU within four hours), and procedure complexity (single- vs. multi-vessel grafting). Nonetheless, the inconsistent and sparse data did not allow for formal subgroup quantitative analyses. There were not many studies (fewer than 10) that gave similar results. Due to the lack of comprehensive research on the subject and our design not presupposing a thorough evaluation of publication bias, we used funnel plots and other statistical experiments, such as Egger regression.

Results

Study Selection

**Figure 1:**
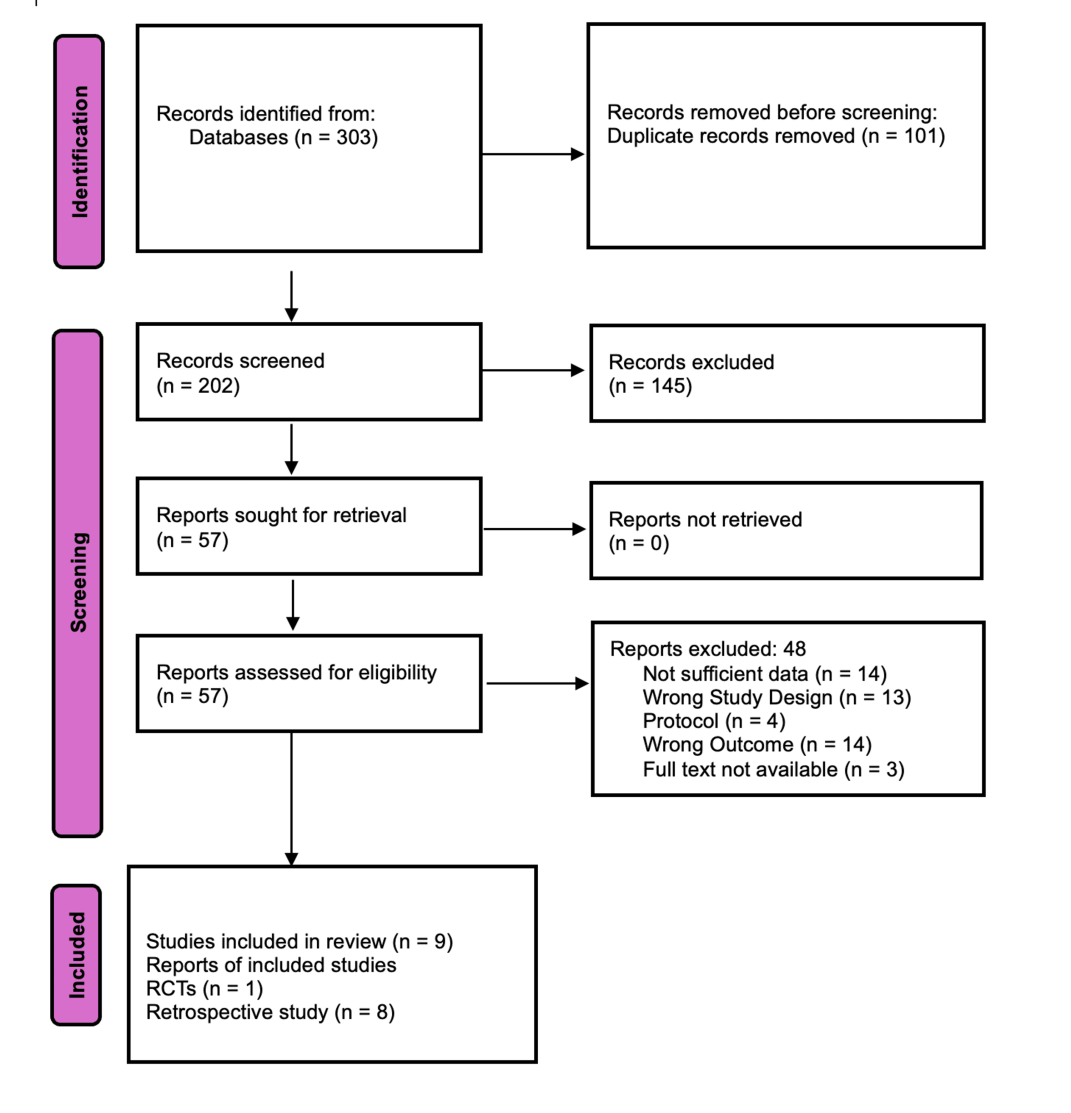
PRISMA flow diagram for systematic review This diagram illustrates the systematic process of identifying, screening, assessing for eligibility, and including studies for the review. PRISMA: Preferred Reporting Items for Systematic Reviews and Meta-Analyses; RCT: randomized controlled trial

The systematic search process and study selection are shown in the PRISMA 2020 flow diagram (Figure 1).

An initial comprehensive search of multiple databases yielded a total of 303 records. After duplicate removal, 202 unique titles and abstracts were screened for relevance. Following this, 57 full-text articles were evaluated against the eligibility criteria. Ultimately, nine studies met all inclusion criteria and were included in the review [20-30].

Risk of Bias Assessment

**Figure 2:**
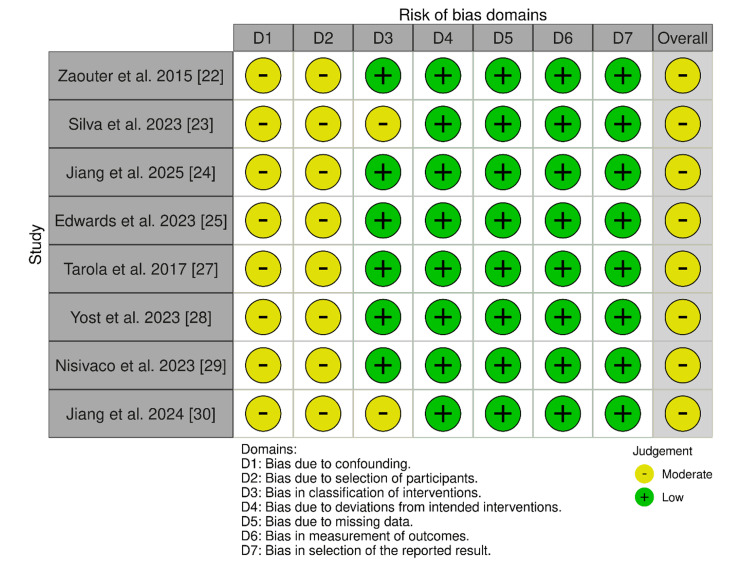
Risk of bias assessment summary from the included observational studies. This diagram presents the specific risk of bias judgments for each of the eight included retrospective cohort studies across all seven methodological domains using the ROBINS-I tool. ROBINS-I: Risk Of Bias In Non-randomized Studies of Interventions References [22-25,27-30]

The risk of bias evaluation of the included observational studies, as assessed by the ROBINS-I tool, is presented in Figure 2.

**Figure 3:**
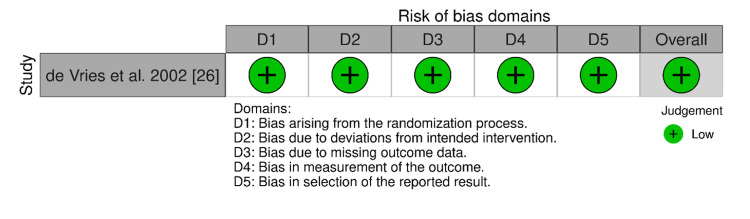
Risk of bias assessment summary from the included randomized control study. This diagram presents the specific risk of bias judgments for the included single randomized controlled trial across all seven methodological domains using the Cochrane Risk of Bias 2 (RoB 2) tool. Reference [26]

The risk of bias in any study was estimated based on these domains by judging the overall risk of bias as low, moderate, or serious. The overwhelming majority of the studies had a moderate risk of bias in the areas of confounding (D1) and selection bias (D2) because of their retrospective and observational methodology and non-random selection of the participants (which could have created imbalances in baseline characteristics). To illustrate, patient selection and group disparity in comorbidities and functional status have raised serious concerns in research papers by Zaouter et al., Tarola et al., and Nisivaco et al [22,27,29]. The risk of bias evaluation of the included RCT study, as assessed by the RoB 2 tool, is presented in Figure 3.

This RCT study by de Vries et al. had a low risk of bias across all domains, reflecting the strength of the prospective randomization and standardized protocols [26].

Characteristics of Included Studies

The studies included in the paper have a diverse international outlook, with research conducted in nations across Europe, North America, Asia, and Latin America. Most of the incorporated studies were retrospective cohort studies, and only one was an RCT. The sample sizes were diverse, ranging from a smaller cohort of 71 patients to a large-scale analysis of 947 patients, totaling 3,223 adult patients. The age groups of the patients were typically homogeneous, and the majority of the studies were devoted to adults with coronary artery bypass surgery (mostly) with single-vessel disease (including LAD revascularization or isolated CABG). The gender distribution was found to be male predominant, ranging between 50% and 85%, as per the cohorts representing normal gender distributions in cardiac surgery patients. The ages were mainly in the sixth decade of life, with the mean and median ages ranging between 60 and 67 years. The literature primarily consisted of studies on robotic or minimally invasive surgery methods. Others paid much attention to types of completely endoscopic coronary artery bypass (TECAB) surgery with robotic assistance, often being off-pump with beating-heart methods to reduce invasiveness and cardiopulmonary bypass-related events [22,25,27,29]. Other procedures were MIDCAB using the thoracoscopic or a little lateral thoracotomy method [26]. There were also studies using more inclusive MICS procedures and hybrid revascularization plans [24,30]. The intervention strategies in the studies focused on UFTE approaches, characterized by the removal of the endotracheal tube at the conclusion of the operation in the OR or within one to four hours of admission to the ICU. These guidelines were frequently used in conjunction with the ERAS pathways, which comprised multimodal analgesia, opioid-sparing regimens, and regional anesthesia methods such as fascial plane blocks or thoracic epidural analgesia. The purpose of these strategies was to facilitate early extubation, minimize exposure to sedation, and enhance postoperative recovery. Comparator arms usually referred to routine postoperative care, which included mechanical ventilation lasting longer than four hours, most often in the ICU. These control groups used regular anesthesia and extubation procedures that the institutions used. The inclusion criteria in the studies were relatively similar and primarily included adult patients undergoing redo sternotomy, those with high surgical risk (e.g., high Society of Thoracic Surgeons (STS) scores, severe comorbidities), or those undergoing emergent surgery. Other studies have specifically observed preserved left ventricular function and a low predicted surgical risk as a prior condition, focusing on patient selection in early extubation practices, which are safety-based [22,24,27,29,30]. The primary evaluated outcomes focused on uniformity and included ICU LOS, POAF incidence, and hospital LOS. These primary outcomes were evaluated in all nine studies that were included. Other secondary outcomes that were assessed included 30-day mortality, re-intubation, perioperative complications, transfusion needs, and readmission [22,23,25,26,28,29]. We present a comprehensive summary of the included studies, detailing the author, year, country, sample size, population, key patient demographics (male percentage, mean/median age), specific robotic surgical approach, the definition of the UFTE intervention, the conventional comparator, and the primary outcomes (Table 1).

**Table 1:** Characteristics of included studies This table provides a comprehensive summary of the included studies, detailing the author, year, country, sample size, population, key patient demographics (male percentage, mean/median age), specific robotic surgical approach, the definition of the ultra-fast extubation (UFTE) intervention, the conventional comparator, and the primary outcomes. TECAB: Totally endoscopic coronary artery bypass, STD: standard/conventional care, UFTCA: ultra-fast-track cardiac anesthesia, CGA: conventional general anesthesia, POD1, POD2: postoperative day 1, 2, ICU: intensive care unit, OR: operating room, LAD: left anterior descending artery, LIMA: left internal mammary artery, IQR: interquartile range, MV: mitral valve, PACU: post-anesthesia care unit, CAD: coronary artery disease, ASA: American Society of Anesthesiologists, NYHA: New York Heart Association

Study (Author, Year)	Country	Study Design	Sample Size (Groups)	Population	Male (%)	Age (Mean/Median)	Surgery Type/Approach	Intervention (Ultra-Fast Extubation Protocol)	Comparator	Inclusion Criteria (Key)	Primary Outcomes (Key)
Zaouter et al., 2015 [22]	France	Retrospective	71 (TECAB: 38, Std CABG: 33)	Single coronary revascularization (LIMA-to-LAD)	TECAB: 86.8% Std CABG: 66.7%	TECAB: 64±10 Std CABG: 67±11	Robotic TECAB (beating heart) vs. Standard CABG (sternotomy, CPB)	Beating-heart TECAB + ERAS, OR extubation	Std CABG + traditional care, ICU extubation (<6h)	LIMA-to-LAD grafting, no previous sternotomy	Feasibility/safety of ERAS, extubation, complications, ventilation, transfusion, ICU/hospital stay
E Silva et al., 2023 [23]	Latin America (Brazil)	Retrospective cohort	402 (OR Extubated: 201, ICU Extubated: 201)	Primary/isolated CABG, ≥18 years	OR Extubated: 76% ICU Extubated: 75%	OR Extubated: 62±8.4 ICU Extubated: 63±9.4	Coronary Artery Bypass Grafting (CABG)	Extubation in the Operating Room (OR)	Extubation in the Intensive Care Unit (ICU)	Primary/isolated CABG, ≥18 years	ICU/hospital LOS, reintubation, morbidity, mortality
Jiang et al., 2025 [24]	China	Retrospective	947 (UFTCA: 439, CGA: 508)	Minimally Invasive Cardiac Surgery (MICS)	UFTCA: 50.8% CGA: 53.9%	UFTCA: 55±23 CGA: 61±16	MICS via right lateral thoracotomy	Ultra-Fast-Track Cardiac Anesthesia (UFTCA) (extubation within 1h post-op), includes fascial plane block, dexamethasone	Conventional General Anesthesia (CGA)	ASA I-III, NYHA I-III, age ≥18	Factors influencing UFTCA, post-op recovery (ICU/hospital LOS, delirium, analgesia, PONV, pulmonary function)
Edwards et al., 2023 [25]	USA	Retrospective	196 (ICU-Bypass: 40, Conv. ICU: 156)	Robotic-assisted single-vessel CABG	ICU-Bypass: 92.5% Conv. ICU: 69.2%	ICU-Bypass: 60.3±9.2 Conv. ICU: 64.2±10.5	Robotic-assisted LIMA-to-LAD CABG	Ultra-fast-track ICU-bypass (PACU/floor recovery, OR extubation)	Conventional ICU recovery	Primary LIMA-to-LAD CABG, specific exclusions	Cost differentials, ICU/hospital LOS, complications
de Vries et al., 2002 [26]	The Netherlands	Randomized prospective	85 (Extubated: 29, Epidural: 28, Intubated: 28)	Elective Minimally Invasive Direct CABG (MIDCAB)	Extubated: 73.3% Epidural: 66.7% Intubated: 83.3%	Extubated: 60±11 Epidural: 57±11 Intubated: 60±12	MIDCAB (LIMA-to-LAD, beating heart)	Immediate extubation + GA + thoracic epidural	Immediate extubation + GA only; vs. GA + post-op ventilation	Elective MIDCAB, no emergency/coagulation issues	Extubation effects, epidural role, hemodynamics, blood gas, pain, hospital stay, oxygenation
Jiang et al., 2024 [30]	China	Retrospective observational	543 (UFTCA: 327, CGA: 216)	Minimally Invasive Cardiac Surgery (MICS)	UFTCA: 50.8% CGA: 51.4%	UFTCA: 55.5 [IQR] CGA: 57 [IQR]	Thoracoscopic-assisted right-side axillary incision MICS	Ultra-fast-track cardiac anesthesia (UFTCA) (extubation immediately or within 1h post-op), includes low-dose opioid, pectoral/serratus plane block	Conventional General Anesthesia (CGA)	ASA I–III, NYHA ≤III, LVDD ≤II, age 18–79	ICU/hospital LOS, postoperative oxygenation, complications
Tarola et al., 2017 [27]	Canada	Retrospective	90 patients	Elective/urgent robotic-assisted CABG (STS <3)	73%	60.99±9.46	Robotic-assisted MICS CABG	Ultrafast track (OR extubation, PACU/ward recovery, bypassing ICU)	(Implicitly traditional ICU care)	STS <3, specific CAD, CT scan; exclusions: unstable angina, high LV grade/creatinine, emergent	In-hospital outcomes (mortality, re-exploration, wound infection, LOS)
Yost et al., 2023 [28]	USA	Retrospective	169 (Early Discharge: 57, Std Discharge: 112)	Robotic cardiac surgery (various procedures)	Early Discharge: 70.2%	Early Discharge: 62 [IQR: 55, 66]	Various robotic cardiac surgeries (MV repair, atrial mass resection, CABG, etc.)	Early discharge (POD1 or POD2), OR extubation, multimodal pain control	Standard discharge (POD3 or later)	Low preoperative risk, uncomplicated post-op course	Morbidity, mortality, 30-day readmission
Nisivaco et al., 2023 [29]	USA	Retrospective, single-center	720 (POD1: 93, Standard: 627)	Robotic beating-heart TECAB (single/multivessel)	POD1: ~85% Standard: ~76%	POD1: 64±11 Standard: 66±10	Robotic beating-heart TECAB	Ultrafast-track for POD1 discharge (early chest tube removal, OR extubation)	Standard discharge (POD2 or later)	Robotic TECAB for sternal-sparing surgery; specific POD1 criteria	Extubation time, chest tube drainage, mortality, transfusion, readmission, return to activity/work, midterm cardiac mortality

Primary Outcome: ICU LOS

Other studies have shown a drastically reduced ICU stay among patients treated on an ultra-fast-track basis [22,24,25,28-30]. Zaouter et al. found that patients undergoing TECAB with an enhanced recovery protocol had a median ICU stay of 21 hours, compared to 45 hours in the standard CABG and fast-track anesthesia group (a 24-hour reduction; p = 0.001) [22]. On the same note, Jiang et al. found that the average stay of patients under ultra-fast-track cardiac anesthesia was 22.8 hours in the ICU, which is significantly lower compared to the 44 hours in the traditional anesthesia group (p < 0.001) [24]. The strongest example of ICU stay reduction is provided by Edwards et al., who found that the implementation of the ICU-bypass protocol resulted in zero ICU stay for successful patients, compared to 30.9 hours of ICU stay in the control group (p < 0.0001) [25]. Even those who had to be converted to ICU care had a lower median ICU time, although not by as drastic a margin. Conversely, E Silva et al. found no significant difference in the ICU LOS between patients who were extubated in the OR and patients who were extubated in the ICU (means around 68-69 hours, p=0.975), indicating that it may be that institutional or procedural effects may have an impact on outcomes in specific institutions [23]. We present the primary outcome data on ICU LOS from the included studies, comparing UFTE protocols with traditional ones based on ventilation (Table 2).

**Table 2:** Primary outcome: intensive care unit length of stay (ICU LOS) This table compares the ICU LOS between the UFT and conventional ventilation groups across the included studies. CABG: coronary artery bypass grafting, PACU: post-anesthesia care unit, POD1, POD2: postoperative day 1, 2

Study (Author, Year)	Intervention Group	Control/Comparison Group	ICU Length of Stay (Intervention Group)	ICU Length of Stay (Control/Comparison Group)	Significance (p-value)	Key Finding/Observation Regarding ICU LOS
Zaouter et al., 2015 [22]	TECAB (Totally Endoscopic CABG) + ERAS	Standard CABG + Fast-Track Cardiac Anesthesia	21 hours (median 19-43)	45 hours (median 28-49)	p = 0.001	Significant 24-hour reduction in ICU stay in the TECAB group.
E Silva et al., 2023 [23]	Extubated in the Operating Room (OR)	Extubated in the ICU	68 ± 32 hours (mean ± SD)	69 ± 31 hours (mean ± SD)	p = 0.975	No significant difference in total ICU length of stay between OR-extubated and ICU-extubated groups.
Jiang et al., 2025 [24]	Ultra-Fast-Track Cardiac Anesthesia (UFTCA)	Conventional General Anesthesia (CGA)	22.83 ± 20 hours (mean)	44 ± 43 hours (mean)	p < 0.001	UFTCA group had a significantly shorter ICU stay in Minimally Invasive Cardiac Surgery (MICS).
Edwards et al., 2023 [25]	ICU-Bypass Protocol	Conventional ICU Recovery	0 hours (for successful candidates)	30.90 hours (median)	p < 0.0001	Successful ICU-bypass completely eliminated ICU duration. "Conversion-to-ICU recovery" group had a slight net reduction in median ICU duration (3.8 hours).
de Vries et al., 2002 [26]	Epidural Group (immediate extubation)	Intubated Group (postoperative ventilation)	All patients (except one) discharged from ICU within 24 hours	Prolonged ICU stay for one patient due to pneumonia	Not directly reported for group comparison	All patients (except one in the intubated group) were discharged from the ICU within 24 hours.
Jiang et al., 2024 [30]	Ultra-Fast-Track Cardiac Anesthesia (UFTCA)	Conventional General Anesthesia (CGA)	23 hours (median 20)	43 hours (median 45)	P = 0.001	UFTCA group demonstrated markedly less ICU stay compared to CGA group, partly due to intentional delays in extubation in the CGA group due to staff shortages.
Tarola et al., 2017 [27]	Ultrafast-Track Robotic-Assisted CABG	(Bypass of cardiac surgical ICU)	Bypassed ICU (recovery in PACU/ward)	One patient admitted to ICU for 17 hours after re-exploration for bleeding.	Not applicable (direct bypass)	Patients were designed to bypass the cardiac surgical ICU entirely. Mean PACU length of stay was 4.5 ± 0.4 hours.
Yost et al., 2023 [28]	POD1 Discharge Group (robotic cardiac surgery)	POD2 Discharge Group	18 hours (median 17-28)	22 hours (median 20-31)	P = 0.04	Shorter ICU duration observed in the POD1 group compared to the POD2 group. Overall median ICU LOS was 22 hours for POD1-2 discharge group.
Nisivaco et al., 2023 [29]	POD1 Discharge Group (robotic TECAB)	Standard Discharge Group (POD2 or later)	24.0 ± 0.0 hours (mean ± SD)	31 ± 16 hours (mean ± SD)	P < 0.001	POD1 discharge group had a significantly shorter ICU length of stay.

Secondary Outcome: POAF Incidence

We present the incidence of POAF in the included studies, comparing UFTE/early extubation protocols with more traditional management (Table 3).

**Table 3:** Postoperative atrial fibrillation (POAF) incidence in cardiac surgery studies The table shows the incidence of POAF across the UFT and conventional management groups. ERAS: enhanced recovery after surgery, N/A: not available; TECAB: totally endoscopic coronary artery bypass; CABG: coronary artery bypass grafting

Study (First Author, Year)	Groups Compared	POAF Incidence (Group 1)	POAF Incidence (Group 2)	POAF Incidence (Group 3)	p-value
Zaouter et al., 2015 [22]	Robotic TECAB + preliminary ERAS vs. Standard CABG + traditional perioperative care	7 (18%) in TECAB group	3 (9%) in Standard CABG group	N/A	0.320
de Vries et al., 2002 [26]	Extubated (General Anesthesia) vs. Epidural (Thoracic Epidural + General Anesthesia) vs. Intubated (General Anesthesia)	2 patients in Extubated group	3 patients in Epidural group	4 patients in Intubated group	Not provided for this comparison
Edwards et al., 2023 [25]	Conventional ICU Recovery vs. Conversion-to-ICU Recovery vs. Successful ICU-Bypass	33 (21.15%) in Conventional ICU Recovery group	2 (10.53%) in Conversion-to-ICU Recovery group	7 (17.50%) in Successful ICU-Bypass group	0.33
E Silva et al., 2023 [23]	Extubated in the Operating Room (OR) vs. Extubated in the ICU	28 (14%) in OR Extubated group	25 (12%) in ICU Extubated group	N/A	0.768
Yost et al., 2023 [28]	Postoperative Day 1 (POD1) discharge vs. Postoperative Day 2 (POD2) discharge	0.0% (0/19) in POD1 group	5.2% (2/38) in POD2 group	N/A	0.80
Nisivaco et al., 2023 [29]	POD1 discharge vs. Standard discharge (POD2 or later)	3 (3.2%) in POD1 discharge group	85 (14%) in Standard discharge group	N/A	0.004

Data indicate that POAF is not significantly increased during early extubation strategies in the majority of the studies. For example, Zaouter et al. reported a POAF rate of 18% in the robotic TECAB group, which included enhanced recovery factors, compared to 9% in patients who received standard CABG; however, this difference was not statistically significant [22]. The randomized trial by de Vries et al. provided POAF rates in patients under general anesthesia, undergoing epidural analgesia, or those requiring postoperative ventilation; no comparative data were given [26]. On the same note, Edwards et al. did not observe a significant difference in POAF in patients treated with traditional ICU recovery, conversion to ICU, or successful ICU bypass; rates ranged from 10% to 21% [25]. In a study by E Silva et al., there was no statistical difference in POAF rates in patients who were extubated in an OR (14%) or in patients whose extubation was done in an ICU (12%) [23]. In Yost et al., there was no POAF in patients discharged on the first postoperative day, and a rate of 5.2% on the second day without a significant difference [28]. Interestingly, Nisivaco et al. also found a significantly lower POAF rate among patients discharged on postoperative day one (3.2%) compared to those discharged later (14%), indicating that early extubation and discharge practices may have a protective influence [29].

Secondary Outcome: Hospital LOS

We summarize the hospital LOS in several studies that compare ultra-fast-track care with conventional care paths (Table 4).

**Table 4:** Hospital length of stay (LOS) in cardiac surgery studies The table summarizes the hospital LOS for patients managed with ultra-fast-track (UFT) pathways versus conventional care. ERAS: enhanced recovery after surgery, N/A: not available; TECAB: totally endoscopic coronary artery bypass; CABG: coronary artery bypass grafting

Study (Author, Year)	Groups Compared	Hospital LOS (Group 1)	Hospital LOS (Group 2)	Hospital LOS (Group 3)	Difference	p-value
Zaouter et al., 2015 [22]	Robotic TECAB + preliminary ERAS vs. Standard CABG + traditional perioperative care	8 days (TECAB group)	12 days (Standard CABG group)	N/A	4 days reduction	<0.001
de Vries et al., 2002 [26]	Extubated (General Anesthesia) vs. Epidural (Thoracic Epidural + General Anesthesia) vs. Intubated (General Anesthesia)	6.6 ± 3.3 days (Extubated group)	5.9 ± 2.4 days (Epidural group)	8.1 ± 5.1 days (Intubated group)	~1.5 days reduction	0.05 (Epidural vs. Intubated)
Edwards et al., 2023 [25]	Conventional ICU Recovery vs. Conversion-to-ICU Recovery vs. Successful ICU-Bypass	5.94 (2.62) days (Conventional ICU Recovery)	4.21 (1.51) days (Conversion-to-ICU Recovery)	4.30 (1.68) days (Successful ICU-Bypass)	~1.6 days reduction	<0.0001 (Overall)
E Silva et al., 2023 [23]	Extubated in the Operating Room (OR) vs. Extubated in the ICU	10 ± 6 days (OR Extubated group)	11 ± 6 days (ICU Extubated group)	N/A	2 days reduction	0.013
Yost et al., 2023 [28]	Postoperative Day 1 (POD1) discharge vs. Postoperative Day 2 (POD2) discharge vs. Standard discharge (POD3 or later)	1 day (POD1 discharge group)	2 days (POD2 discharge group)	4 days (Standard discharge group)	At least 2 days reduction	Not specified for POD1/2 vs POD3+, but notes similar 30-day readmission/mortality
Nisivaco et al., 2023 [29]	POD1 discharge vs. Standard discharge (non-POD1 discharge)	1.0 ± 0.0 days (POD1 discharge group)	2.57 ± 0.76 days (Standard discharge group)	N/A	~1.57 days reduction	<0.001
Jiang et al., 2025 [24]	Ultra-Fast-Track Cardiac Anesthesia (UFTCA) vs. Conventional General Anesthesia (CGA)	8 ± 3 days (UFTCA group)	10 ± 5 days (CGA group)	N/A	2 days reduction	<0.001
Jiang et al., 2024 [30]	UFTCA vs. CGA	8 days (UFTCA group)	10 days (CGA group)	N/A	2 days reduction	0.001
Tarola et al., 2017 [27]	Ultrafast Track Robotic-Assisted Minimally Invasive Coronary Artery Surgical Revascularization	3.5 ± 1.17 days (Ultrafast Track group, no direct comparator in table)	N/A	N/A	N/A	N/A

A reduced period of hospital stay due to early discharge or extubation procedures was a steady trend. Zaouter et al. reported that patients who underwent robotic TECAB using improved recovery guidelines stayed in the hospital for a median of eight days, compared to 12 days in the standard CABG group, representing a four-day difference [22]. De Vries et al. observed that the mean hospital stay decreased by 1.5 days in patients who were extubated immediately or received epidural analgesia compared to those who required postoperative ventilation [26]. Edwards et al. [25] reported that patients treated with ICU bypass or conversion protocols had a shorter hospital LOS, approximately 1.6 days, compared to standard ICU recovery. Similarly, the results of E Silva et al. showed a two-day reduction in hospital stay for patients who were extubated in the OR compared to those who were extubated later in the ICU [23]. Yost et al. noted that, on discharge during postoperative days 1 and 2, the hospital stays of patients were significantly lower, with median values of one and two days, respectively, compared to the four days associated with standard discharge at postoperative day three and above [28]. The same was reported by Nisivaco et al., who found a decrease in hospital stay of 1.57 days for patients discharged on postoperative day 1 compared to those discharged later [29]. Jiang et al. corroborated these results in their cohorts and found a linear two-day reduction in hospitalization time for ultra-fast-track cardiac anesthesia compared to conventional anesthesia [30]. In ultra-fast-track robotic CABG patients, Tarola et al. reported an average hospital stay of 3.5 days, but did not provide a comparator group [27].

Additional Outcomes

Analysis of secondary safety and recovery outcomes revealed that the UFTE protocols did not increase the risk of major complications. Re-intubation rates were low (0-5%), and there was no significant difference between UFTE and conventional groups [22-24,26]. On the same note, 30-day and in-hospital mortality rates were insignificant (0-1%) in all the study cohorts [22,25,27-29]. It is also worth noting that UFTE was associated with multiple positive recovery measures. One of the recurring results was a decrease in blood transfusion rates, with UFTE groups recording a range of 0-24% compared to the conventional groups, which ranged from 12% to 55% [22,25,29]. Moreover, readmission rates for perioperative UFTE and early discharge practices were either equal to or lower than those of other practices [28,29]. In addition to the use of resources, the UFTE protocols that involved the use of multimodal, opioid-sparing methods like fascial plane blocks and thoracic epidurals led to better pain management and decreased postoperative opioid use by a significant margin [24,26,27]. This enhanced recovery was also supported by an indication of an earlier return to work and daily tasks of patients under UFTE pathways [29]. A summary of these other outcomes is displayed in Table 5.

**Table 5:** Summary of additional outcomes extracted from the included studies The table presents data on critical secondary safety and recovery outcomes beyond the primary endpoints. UFTE: ultra-fast-track extubation

Outcome	Finding (UFTE vs. Conventional)	Supporting Studies
Re-intubation	No significant difference; rates very low (0-5%)	Zaouter et al., 2015 [22]; E Silva et al., 2023 [23]; Jiang et al., 2025 [24]; de Vries et al., 2002 [26]
Mortality	No significant difference; rates very low (0-1%)	Zaouter et al., 2015 [22]; Edwards et al., 2023 [25]; Tarola et al., 2017 [27]; Yost et al., 2023 [28]; Nisivaco et al., 2023 [29]
Blood Transfusion	Lower in UFTE groups (e.g., 0-24% vs. 12-55%)	Zaouter et al., 2015 [22]; Edwards et al., 2023 [25]; Nisivaco et al., 2023 [29]
Readmission	Comparable or lower in UFTE/early discharge groups (e.g., 3.2% vs. 5.7%)	Yost et al., 2023 [28]; Nisivaco et al., 2023 [29]
Pain and Opioid Use	Improved pain control and reduced opioid consumption with UFTE protocols	Jiang et al., 2025 [24]; de Vries et al., 2002 [26]; Tarola et al., 2017 [27]
Return to Work	Earlier return to work/activity demonstrated in studies that reported this outcome.	Nisivaco et al., 2023 [29]

Discussion

This systematic review summarized existing data on the clinical effects of UFTE, defined as extubation with four hours of postoperative ventilation or in the OR, compared to traditional postoperative ventilation after RA-CABG. The main conclusion is that UFT protocols consistently result in a significant decrease in LOS in an ICU, with reported lengths of stay ranging from 4 to 30 hours per study. In line with this, LOS in the hospital was significantly shorter for patients who underwent UFT extubation, with an average reduction of one to four days. Critically, patient safety was not lost in UFT because similar rates of postoperative complications, such as atrial fibrillation (POAF), re-intubation, stroke, and mortality, were revealed to be identical to those of the study areas. Several investigations observed tendencies towards reduced POAF rates and fewer blood transfusions, which may also be indicators of improved perioperative physiology.

These outcomes build upon and supplement the existing evidence on which case, primarily based on standard or standard-open-heart surgery populations, fast-track and UFTE protocols have been demonstrated to decrease the magnitude of resource use without an increase in adverse event rates [31-33]. The decreased mechanical ventilation time is achievable and effective in accelerating recovery and reducing ICU stay in this subset of robotic surgical operations, which includes my least invasive oncology procedures featuring microsurgical techniques, limited incisions, and minimal tissue trauma. The fact that the decrease in ICU LOS observed in the robotic and conventional groups was similar supports the ability to generalize the principles of fast-track anesthesia in different surgical varieties.

The described neutral or beneficial result on POAF is in line with the mechanistic considerations, which implicate decreased sympathetic activation, mitigated systemic inflammation, and minimized sedative exposure as mediators of arrhythmia alleviation [17,34]. Although the rates of POAF did not statistically differ between UFT and conventional ventilation in most studies, the overwhelming decrease reported by Nisivaco et al. [29] warrants attention and further examination. This is specifically true because POAF is a frequent complication that is not only morbid but also pervasive during extended ICU stay and resource use [34-36]. Lower incidences of re-intubation (<5%), negligible mortality, and comparable readmissions enhanced the safety aspect of UFT extubation after RA-CABG. This encouraging fact has justified the rationality of these guidelines when implemented in well-chosen, low-risk patients and in facilities equipped with qualified multidisciplinary teams and uniform perioperative algorithms. These results were likely influenced by the introduction of the ERAS principles, which focus on the use of multimodal analgesia, opioid-sparing measures, and early mobilization, as previously reported in cardiac surgical populations [12,37-39].

There are, however, several significant limitations that should be acknowledged, despite these promising results. The total body of evidence has been limited by a higher proportion of single-center, retrospective observational studies with moderate risks of bias, primarily due to patient selection and confounding. The majority of cohorts involved less risky subjects whose left ventricular function is well preserved and have few comorbidities, which restricts the external validity to those at very high risk or more complicated surgery candidates. Extrapolation is challenging due to the heterogeneity of surgical techniques, including the use of TECAB, MIDCAB, and variable cardiopulmonary bypass, as well as a combination of these techniques. Moreover, there is significant variability in the methodology used to define UFT protocols, with inconsistent timing limits and varying perioperative anesthetic regimens.

Inequalities in data reporting preclinically hindered the formal meta-analysis; most studies reported results in median values without standard deviations or used different time units for ICU and hospital LOS. This is because the number of RCTs is minimal, with only one relatively small RCT [26] available, which limits the causal inferences that can be made. The overwhelming majority of the data came from observational research, which, even with propensity matching or multivariate adjustment, cannot eliminate residual confounding.

Another possible issue is publication bias. Research resulting in positive evidence regarding new robotic and UFT regimes will be published more frequently, while negative or neutral results may remain hidden. Additionally, the majority of single-surgeon or single-institution experiences can overestimate efficacy estimates due to the expert knowledge and selection biases inherent in these studies. Although authors were approached to clarify their missing information, incomplete/unpublished findings may also influence generalizability.

Clinically and in the role of a nurse, this review can be used to justify the incorporation of UFT extubation into the overall care of the perioperative phase of robotic cardiac surgery. Nurses are central to the adoption of fast-track extubation paths, and they require skills in speedy patient evaluation, vigilant airway observation, sedation control, and promoting rapid movement [12,40]. The education of perioperative and ICU nursing personnel should focus on the individual physiological aspects of robotic surgery patients, including the early detection of the patient's readiness to be weaned from the ventilator, and the prevention of postoperative complications [12]. The collaboration between nurses, intensivists, anesthesiologists, and surgeons is required in a multidisciplinary setting that maximizes the result [24].

UFT protocols have been implemented in accordance with healthcare policy requirements to enhance efficiency and reduce costs. Reduced ICU use and LOS translate into direct cost savings and increased capacity, which is crucial in cardiac centers operating under resource constraints as they manage large volumes of patients [33]. Standardized pathways, when adopted by other institutions, can also decrease the variability of outcomes and enhance the quality of care [41].

The implications for research are that future studies should focus on large-scale multicenter RCTs involving UFT extubation use among well-characterized robotic CABG patients, with defined interventions and comprehensive outcome measures. Long-term assessment of functional outcomes, patient-reported outcomes, and cognitive assessments will provide significant information in addition to traditional clinical measures. In addition, rigorous cost-effectiveness studies that encompass both direct and indirect healthcare expenditures would be justified to inform the allocation of resources. Clinical trials to explore the relationship between UFT extubation, anesthetic depth, and arrhythmia pathways have the potential to clarify how to further improve the risk mitigation of POAF.

New data indicate the possible advantages of regional anesthesia methods and opioid-sparing regimes in shortening the time taken to extubate a person and increasing recovery rates following cardiac surgery. Regional anesthesia has also been found to decrease narcotic use during surgery and facilitate easy extubation, which leads to better patient recovery [42,43]. It is one of the components of a balanced approach to anesthetics, which reduces the consumption of opioids and also contributes to a quicker recovery without decreasing patient safety and comfort [44]. Future studies on optimizing perioperative multimodal analgesic protocols in robotic cardiac surgery, balancing analgesia with timely respiratory recovery, are warranted.

This systematic review reveals growing evidence suggesting that UFTE following RA-CABG may be associated with a significant reduction in ICU and hospital time without apparent harm to patient safety. Nevertheless, since the majority of included studies are of moderate risk of bias and observational in nature, conclusions have not yet been drawn. Such results suggest a robust approach to achieving greater recovery in robotic cardiac surgery, indicating the need to pursue additional high-quality randomized studies that quantify efficacy and safety more definitively. It is possible that educational interventions that focus on nursing and multidisciplinary teams can enhance the uptake and sustainability of fast-track care models.

## Conclusions

UFTE post robotic-only surgery, CABG, is accompanied by reduced ICU and hospital LOS without adverse patient outcomes, reflected in no postoperative complications such as atrial fibrillation. This result confirms the viability of UFTE in the context of enhanced recovery in carefully selected patients with RA-CABG. Nonetheless, a significant portion of the observational evidence required higher-quality randomized trials to confirm these advantages. Extubation with UFTE after RA-CABG offers clear benefits in reducing ICU and hospital stays without increasing risks like POAF. These findings support the integration of UFTE into enhanced recovery protocols for low-risk patients. However, the predominance of observational data underscores the need for larger RCTs to confirm causality and generalizability. The unique aspects of robotic procedures, such as prolonged operative times, do not appear to compromise the safety of early extubation when patient selection is optimized. However, ongoing research should address variations in anesthetic techniques and institutional factors to broaden the applicability of this approach.

## Appendices

Search String PUBMED: ("coronary artery bypass graft*" OR CABG OR "coronary bypass" OR "myocardial revascularization") AND ("robotic*" OR "robot-assisted" OR "minimally invasive" OR MIDCAB OR TECAB OR "thoracoscopic" OR "endoscopic") AND ("early extubation" OR "fast-track" OR "ultra-fast-track" OR "accelerated extubation" OR "shortened ventilation" OR "fast track anesthesia" OR "fast-track recovery" OR "enhanced recovery after surgery" OR ERAS) Found: 26

Embase ('coronary artery bypass'/exp OR 'coronary artery bypass graft*' OR cabg OR cags OR cag) AND ('robot'/exp OR 'robot*' OR 'robot-assisted' OR 'minimally invasive' OR midcab OR tecab OR 'endoscopic' OR 'thoracoscopic') AND ('early extubation' OR 'fast-track' OR 'ultra-fast-track' OR 'accelerated extubation' OR 'shortened ventilation' OR 'enhanced recovery' OR eras) Found: 89

Scopus: TITLE-ABS-KEY("coronary artery bypass" OR CABG) AND TITLE-ABS-KEY(robotic OR "robot-assisted" OR "minimally invasive" OR MIDCAB OR TECAB OR endoscopic OR thoracoscopic) AND TITLE-ABS-KEY("early extubation" OR "fast track" OR "fast-track" OR "ultra-fast-track" OR "accelerated extubation" OR "shortened ventilation" OR "enhanced recovery" OR ERAS) Found: 165

20 Trials matching ("coronary artery bypass" OR CABG OR "myocardial revascularization") AND (robotic OR "robot-assisted" OR "minimally invasive" OR MIDCAB OR TECAB OR endoscopic OR thoracoscopic) AND ("early extubation" OR "fast track" OR "fast-track" OR "ultra-fast-track" OR "accelerated extubation" OR "shortened ventilation" OR "enhanced recovery" OR ERAS) in Title Abstract Keyword - (Word variations have been searched)
